# Subfunctionalization of Parental Polyamine Oxidase (PAO) Genes in the Allopolyploid Tobacco *Nicotiana tabacum* (L.)

**DOI:** 10.3390/genes14112025

**Published:** 2023-10-30

**Authors:** Péter Benkő, Nikolett Kaszler, Katalin Gémes, Attila Fehér

**Affiliations:** 1 Institute of Plant Biology, HUN-REN Biological Research Centre, 62. Temesvári Krt., H-6726 Szeged, Hungary; benko.peter@brc.hu (P.B.) kaszler.nikolett@brc.hu (N.K.); gemeskatalin80@gmail.com (K.G.); 2 Doctoral School of Biology, University of Szeged, 52. Közép Fasor, H-6726 Szeged, Hungary; 3 Department of Plant Biology, University of Szeged, 52. Közép Fasor, H-6726 Szeged, Hungary

**Keywords:** polyamine oxidase, tobacco, homeologous genes, gene expression profile, abiotic stress, phytohormones

## Abstract

Polyamines play an important role in developmental and environmental stress responses in plants. Polyamine oxidases (PAOs) are flavin-adenine-dinucleotide-dependent enzymes associated with polyamine catabolism. In this study, 14 genes were identified in the tobacco genome that code for PAO proteins being named based on their sequence homology with Arabidopsis PAOs (AtPAO1-5): NtPAO1A-B; NtPAO2A-C, NtPAO4A-D, and NtPAO5A-E. Sequence analysis confirmed that the *PAO* gene family of the allopolyploid hybrid *Nicotiana tabacum* is not an exact combination of the *PAO* genes of the maternal *Nicotiana sylvestris* and paternal *Nicotiana tomentosiformis* ones. The loss of the *N. sylvestris* homeolog of *NtPAO5E* and the gain of an extra *NtPAO2* copy, likely of *Nicotiana othophora* origin, was revealed. The latter adds to the few pieces of evidence suggesting that the paternal parent of *N. tabacum* was an introgressed hybrid of *N. tomentosiformis* and *N. othophora*. Gene expression analysis indicated that all 14 *PAO* genes kept their expression following the formation of the hybrid species. The homeologous gene pairs showed similar or opposite regulation depending on the investigated organ, applied stress, or hormone treatment. The data indicate that the expression pattern of the homeologous genes is diversifying in a process of subfunctionalization.

## 1. Introduction

Polyamines are small aliphatic molecules that are involved in numerous developmental processes in plants, such as cell division and organ development [1], pollen growth [2], organogenesis [3], fruit ripening [4], and senescence [5], as well as in coping with various abiotic stresses [4,6,7,8,9,10]. In plants, the major polyamines are the diamine putrescine (Put), the triamine spermidine (Spd), the tetramine spermine (Spm), and the thermospermine (T-Spm) [1,11,12]. Intracellular polyamine homeostasis is controlled by biosynthesis, transport, and catabolism [13,14,15,16,17]. Two enzymes are involved in polyamine catabolism: copper-containing amine oxidase, which catalyzes the oxidation of Put into 4-aminobutanal with the production of NH_3_ and hydrogen peroxide (H_2_O_2_), [18,19]; and the flavin adenine dinucleotide (FAD)-dependent polyamine oxidases (PAOs), which catalyze the oxidative deamination of Spd, Spm, and/or their derivatives either using terminal catabolism (TC) or using backconversion (BC) pathways. Terminal oxidation of Spd and Spm is specifically activated extracellularly and results in H_2_O_2_, 1,3-diaminopropane (DAP), and 4-aminobutanal (in the case of Spd catabolism) or N-(3-aminopropyl)-4-aminobutanal (in the case of Spm catabolism) [20]. Known TC-type PAOs involve ZmPAO1 in maize and OsPAO6 and OsPAO7 in rice. The BC pathway occurs intracellularly, in the cytoplasm or in the peroxisomes, where PAOs convert Spm (or T-Spm) to Spd and/or Spd into Put. All five *Arabidopsis thaliana* PAOs (AtPAO1-5) and seven tomato SlPAOs (SlPAO1-7) have been documented to catalyze BC reactions [14,21].

Common to all BC- and TC-type reactions is the production of H_2_O_2_ [20,22]. It is suggested that PAOs are involved in abiotic stress responses. In Arabidopsis, under heat stress, production of H_2_O_2_ using peroxisomal AtPAO3 was shown to affect the HSP90 heat stress protein [23], while over- or under-expression of *ZmPAO1* in tobacco [24] affected the plant’s thermotolerance [24]. Under drought stress, in *Vitis vinifera*, the H_2_O_2_ produced by PAO activity was shown to regulate ABA-induced stomatal closure [25].

Phylogenetic analysis has categorized plant PAOs into five clades: I, II, III, IV and V [26]. Clade I members, including Arabidopsis AtPAO1, are localized in the cytoplasm and catabolize PAs in the BC reaction pathway. AtPAO5 and supposedly all members in clade III are also cytoplasmic and prefer T-Spm as a substrate catalyzing the backconversion of T-Spm into Spd. Clade IV members also degrade polyamines in the BC pathway, but are localized in the peroxisomes [14,21,27]. Clade V is composed of sequences of monocotyledonous species and still has BC activity. In contrast, clade II members are predominantly from monocotyledonous plant species, catalyzing the oxidation of polyamines using the TC pathway and localized in the apoplast or vacuole [14,21,27,28].

Tobacco (*Nicotiana tabacum*) is one of the most important economic crops belonging to the Solanaceae family and is used as a model plant in the research of tissue culture, plant regeneration, and other related fields. *Nicotiana tabacum* originated in the hybridization of *Nicotiana sylvestris* (S-genome) and *Nicotiana tomentosiformis* (T-genome). It is an allotetraploid species (2n = 4x = 48), with a genome size of 4.5 Gbp containing several repetitive sequences [29]. Gene duplication, including polyploidization, is an important mechanism of the species evolution [30]. In most cases, having two identical genes is not advantageous for a species, so one of the copies is usually lost or becomes a pseudogene. However, if the extra gene product is beneficial to the organism, or if one of the copies is kept as a “backup”, then the two copies may be maintained. Additionally, these two copies can be used by evolution to increase genetic complexity via subfunctionalization or neofunctionalization. Subfunctionalization involves the two gene copies sharing some of the functions of the original gene, while neofunctionalization involves one of the genes gaining a completely new function due to mutations. For duplicate genes to acquire a new function, it may take millions of generations. Polyploidization of *Nicotiana tabacum* took place approximately 400,000–800,000 or even 200,000 years ago [29,31], which can be considered a very recent event at an evolutionary scale. In agreement, a leaf transcriptomic study revealed little evidence of neofunctionalization of the parental genes in tobacco [32]. However, complex changes in gene expression were observed, which could have arisen from gene loss, gene silencing, or subfunctionalization [32].

To reveal the fate of parental *PAO* genes in the hybrid tobacco *Nicotiana tabacum*, we identified all its *PAO* genes, as well as the seven *PAO* genes of *N. sylvestris* and *N. tomentosiformis*, respectively. After carrying out sequence analysis, we studied the expression pattern of the 14 tobacco *PAO* genes, which allowed us to examine the subfunctionalization of the original genes during tobacco’s brief evolutionary trajectory.

## 2. Materials and Methods

### 2.1. Plant Materials, Growth Condition, and Stress Treatments

For the gene expression experiments, wild type *N. tabacum* L. cv. Wisconsin 38 plants were used. Seeds were surface-sterilized and then germinated on agar plates containing a half-strength MS medium [33] including B5 vitamins (Duchefa Biochemie B.V., Haarlem, The Netherlands), 1% sucrose (Molar Chemicals, Halásztelek, Hungary), and 0,6% plant agar (Duchefa Biochemie). The medium was set to pH 5.7–5.8 with 1M KOH and autoclaved. The plants were grown for two weeks at 22 °C and 50% relative humidity in a plant chamber (Fitoclima S 600 PLH, Aralab, Rio de Mouro, Portugal) under long-day conditions (16/8 h photoperiod), and then various abiotic stress treatments were applied. To induce salt stress, the plants were treated with 150 mM NaCl (Molar Chemicals). For temperature stresses, the seedlings were transferred into a water bath at 42 °C (heat) for 5 hours or to a cold room at 4 °C (cold) for 16 hours. Oxidative stress was induced using H_2_O_2_ (5 mM; VWR Chemicals, Vienna, Austria) for three hours. For phytohormone treatments, the leaves of five-week-old plants were treated in petri dishes (9 cm in diameter) using hormone-supplemented potassium buffer (50 mM; pH 6.5) for three hours under light. The hormones were 10 µM indole-3-acetic acid (IAA) (Duchefa Biochemie), 100 µM abscisic acid (ABA) (Sigma-Aldrich, St. Louis, MO, USA), 10 µM kinetin (Sigma-Aldrich), and 10 µM gibberellic acid (GA3) (Sigma-Aldrich). For gene expression analysis, samples were harvested and snap-frozen in liquid nitrogen and stored at −80 °C until usage.

### 2.2. RNA Extraction and Quantitative Real-Time Polymerase Chain Reation (qRT-PCR) Analysis

For total RNA extraction, the Quick-RNA Miniprep Kit (Zymo Research, Irvine, CA, USA) was used, including reagents to remove any contaminating genomic DNA. A NanoDrop™ 2000/2000c spectrophotometer (Thermo Fisher Scientific, Waltham, MA, USA) was used to evaluate the quality and quantity of the total isolated RNA, considering the ideal absorbance ratio (1.8 ≤ A260/280 ≤ 2.0). Of the total RNA, 600 ng was reverse-transcribed for 60 min at 42 ^ο^C and for 10 min at 75 ^ο^C in a 20 µL reaction volume using the RevertAid First Strand cDNA Synthesis Kit (Thermo Fisher Scientific) according to the manufacturer’s instructions. The cDNA products were diluted 1:10 in AccuGENE^®^ water (Lonza, Verviers, Belgium). The primers were designed using the NCBI (https://www.ncbi.nlm.nih.gov, accessed on 5 June 2022) primer design tool [34] and synthesized by Biocenter Ltd. (Szeged, Hungary). The primer sequences are shown in Supplementary Table S1. The relative mRNA levels were determined using qRT-PCR. As reference genes, *Nt Actin-97-like* (LOC107804820) and the ribosomal protein-coding *L25* gene (L18908) were used. The qRT-PCR reactions were carried out using the CFX384 Touch Real-Time PCR Detection System (BioRad Laboratories Inc., Hercules, CA, USA). The PCR mixture contained (in a total volume of 7 µL) 1 µL cDNA, 0.21 µL of the forward primer, 0.21 µL of the reverse primer, and 3.5 µL of Maxima SYBR Green/ROX qPCR Master Mix (2×) (Thermo Fisher Scientific). The reaction mixtures were aliquoted into Hard-Shell^®^ 384-well plates (thin-wall, skirted, clear/white; Bio-Rad Laboratories Inc., Cat. no: HSP3805). For amplification, a standard two-step thermal cycling profile was used (10 s at 95 °C and 1 min at 60 °C) during 40 cycles, after a 15 min preheating step at 95 °C. Finally, a dissociation stage was added at 95 °C for 15 s, 60 °C for 15 s, and 95 °C for 15 s. The data analysis was performed using the Bio-Rad CFX Maestro (Bio-Rad) software and Microsoft Excel 2016. The relative mRNA levels were calculated using the 2^−∆∆Ct^ method. Data were averaged from three independent biological experiments with three technical replicates for each gene/sample combination.

### 2.3. Statistical Analysis

Statistical analysis was performed using the SIGMAPLOT 12.0 statistical software. Quantitative data are presented as the mean ± SE and the significance of difference between sets of data was determined using one-way analysis of variance (ANOVA) following Duncan’s multiple-range tests; *p*-values of less than 0.05 were considered significant. For pairwise comparisons, Student’s *t*-test was used (* *p* ≤ 0.05, ** *p* ≤ 0.01, *** *p* ≤ 0.001).

### 2.4. Sequence Analysis and Bionformatic Tools

All putative PAO genes and cDNAs of the *N. tabacum* cultivars TN90 (whole-genome shotgun sequencing project PRJNA319578; [35]) and K326 (genome sequencing and assembly project PRJNA376174; [29]) were identified using the reported PAO protein sequences of Arabidopsis as queries with tblastn at the National Center for Biotechnology Information (NCBI) site (https://www.ncbi.nlm.nih.gov/datasets/genome/?taxon=4097; last accessed on 1 September 2023) [36]. The conserved motifs in the PAO proteins were identified using the MEME motif-based sequence analysis tool (https://meme-suite.org/meme/tools/meme; last accessed on 1 September 2023) [37]. The *N. sylvestris* (strain TW136, genome sequencing and assembly project PRJNA182500), *N. tomentosiformis* (genome sequencing and assembly project PRJNA182501), and *Nicotiana otophora* (genome sequencing and assembly project PRJNA208212) PAO-coding gene sequences were obtained in a similar way using the *N. tabacum* TN90 PAO gene sequences as queries and BLASTN as the search algorithm. Sequences with a high query coverage value (above 50%) and expect value (above 1e-150) were selected and used in a blastx search for homologs of the encoded proteins in other plant species (Viridiplantae). Seven distinct coding sequences for PAO-like proteins were identified for all three diploid Nicotiana species, respectively. The genomic sequences were compared and their similarity was visualized using Circoletto [38] by running blastn with the parameters -F F -e 1e-30 -v 200 -b 200.

Nucleic acid and/or protein evolutionary analyses and the construction of phylogenetic trees were all performed using the Molecular Evolutionary Genetics Analysis Version 11 (MEGA11) software package [39]. Sequence alignments were generated using the ClustalW algorithm [40]. The evolutionary history was inferred using the Neighbor-Joining method [41]. The optimal trees are presented. Where a scale bar is shown, the trees are drawn to scale, with branch lengths in the same units as those of the evolutionary distances used to infer the phylogenetic tree. The evolutionary distances were computed using the Poisson correction method [42] and are in the units of the number of amino acid substitutions per site. All ambiguous positions were removed for each sequence pair (pairwise deletion option).

The subcellular localization of the NtPAO proteins was predicted using WoLF PSORT [43] (http://www.genscript.com/psort/wolf_psort.html; last accessed on 1 September 2023).

## 3. Results

### 3.1. The Nicotiana tabacum (L.) Genome Contains Fourteen PAO-Coding Genes

The amino acid sequences of the five *A. thaliana* PAO proteins (AtPAO1-5; Supplementary Table S2) were used to query the sequence data of the *N. tabacum* cultivar TN90 whole-genome shotgun sequencing project (PRJNA319578 [35]) and the *N. tabacum* cultivar K326 genome sequencing and assembly project (PRJNA376174 [29]), respectively, at the NCBI site (https://blast.ncbi.nlm.nih.gov/Blast.cgi; last accessed on 31 August 2023) using the tblastn algorithm.

**Figure 1:**
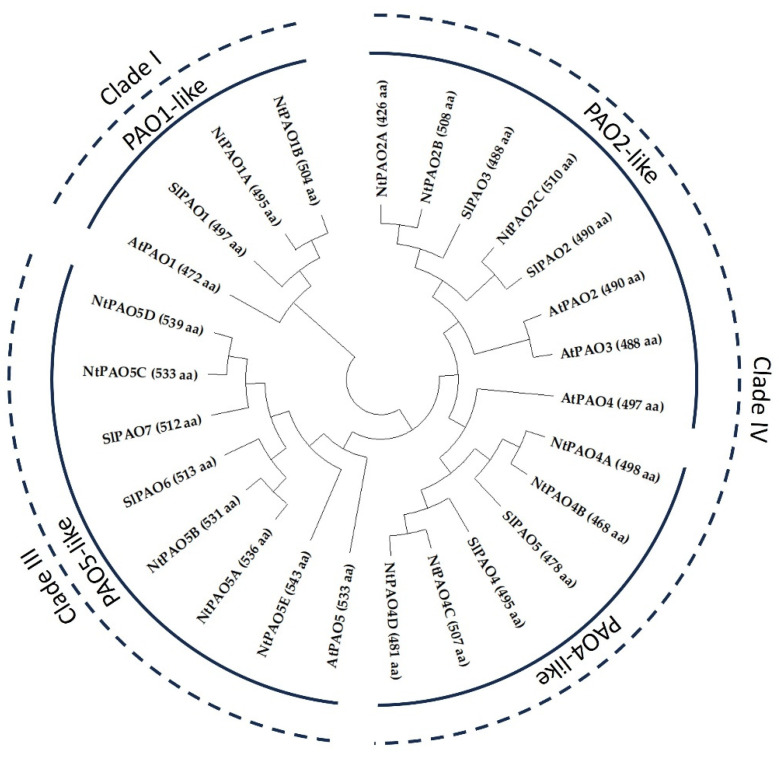
Unrooted phylogenetic tree of *Nicotiana tabacum* (Nt), *Solanum lycopersicum* (Sl), and *Arabidopsis thaliana* (At) PAO protein sequences. The tree was inferred using the Neighbor-Joining method [41]. The optimal tree is shown. The three PAO clades characteristic for dicotyledenous plants [26] are indicated by dashed lines. The four groups widely referred in the scientific literature and named after the Arabidopsis proteins are highlighted using solid lines.

Altogether, 14 PAO-coding genes/cDNAs were identified (Table 1). These were named based on the sequence homology of the coded proteins with Arabidopsis PAOs in agreement with the annotation of the genes as NtPAO1, NtPAO2, NtPAO4, and NtPAO5 (Figure 1 and Supplementary Figure S1A). Although the PAO proteins of dicotyledonous plants represent three of the five plant PAO clades [26], they are rather divided into four groups with the related AtPAO2- and AtPAO4-like proteins separated into two groups (Figure 1). Note that the groups are named after the five Arabidopsis proteins, where AtPAO2 and AtPAO3 are paralogous and fall into the same group (PAO2-like). To keep consistency with the Arabidopsis numbering and the widely used classification of dicotyledonous plant PAOs into four groups, none of the tobacco PAOs were specified as NtPAO3 since none of *N. tabacum* PAOs showed higher similarity to AtPAO3 than to AtPAO2.

The Arabidopsis and tobacco PAO proteins share nine evolutionarily conserved motifs identified by the MEME motif discovery tool, further supporting their structural similarity (Supplementary Figure S1B). Based on the presence of a peroxisomal target sequence (S/A/C)(K/R/H)(L/M) in their C-termini, we suggest that the NtPAO4 and NtPAO2 proteins are localized in the peroxisome, similarly to their Arabidopsis homologs (Supplementary Figure S1C) [14].

**Figure 2:**
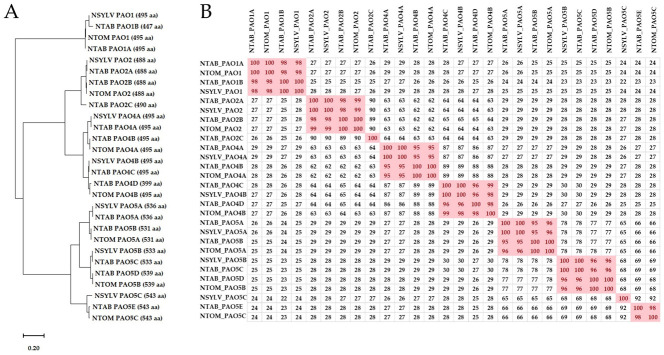
(**A**). Unrooted Neighbour-Joining phylogenetic tree of all PAO protein sequences of *Nicotiana tabacum* (NTAB; for accession codes, see Table 1), *Nicotiana sylvestris* (NSYLV), and *Nicotiana tomentosiformis* (NTOM) (for accession codes, see Supplementary Table S3). (**B**). Amino acid sequence similarity (%) of the same proteins. Similarities at or above 95% are shaded red. aa = amino acid.

**Figure 3:**
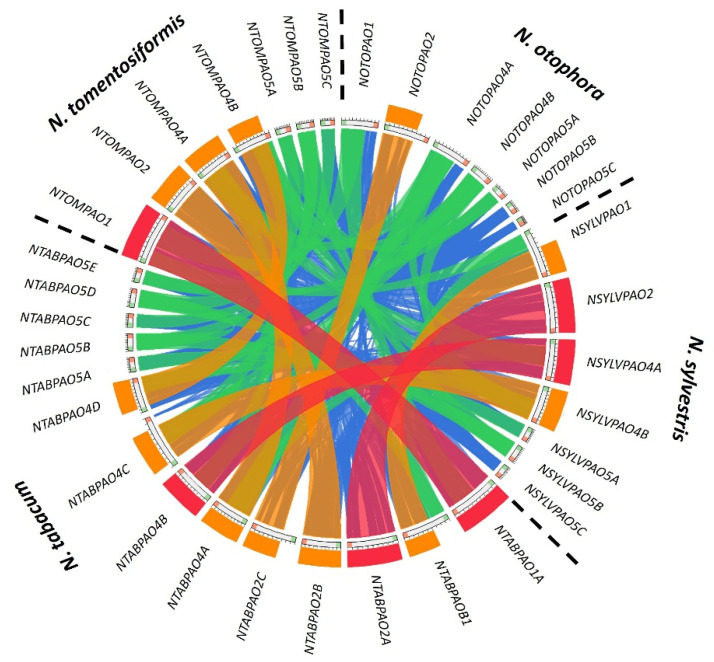
Circos diagram to illustrate the relation of Nicotiana *PAO* gene sequences. The analysis was made using Circoletto based on a comparison of the sequences using blastn [38]. Ribbons are coloured according to bitscores. The ribbons with the highest scores are at the top. The colouring is relative using the “score/maximum” ratio (blue ≤ 0.25, green ≤ 0.50, orange ≤ 0.75, red > 0.75). The minimum and maximum bitscores were 130 and 12002, respectively. Dashed lines separate the genes of the four investigated species.

As compared to the diploid species *Solanum lycopersicum*, which has 7 PAO-coding genes [21], the tetraploid tobacco has 14 coding genes for PAO enzymes. Nevertheless, while tobacco has exactly a double of the tomato set, it has three *PAO2* gene sequences and five *PAO5* sequences, while tomato has two and two, respectively (Figure 1), indicating that the reorganization of *PAO* genes continued during the evolution of Solanaceae. *N. tabacum* is considered to be a hybrid of the ancestors of *N. sylvestris* (S-genome) and *N. tomentosiformis* (T-genome) [31]. As the available genome assembly is just 64% completed [29], localization of the 14 tobacco *PAO* genes to the chromosomes of the S or T genomes was not possible. In order to determine the parental origin of the tobacco *PAO* genes, the *N. tabacum PAO* sequences were compared to the *N. sylvestris* and *N. tomentosiformis* ones [44]. Seven PAO-coding genes were identified in both diploid species (Supplementary Table S3): one *NtPAO1*-like, one *NtPAO2*-like, two *NtPAO4*-like, and three *NtPAO5*-like. The amino acid sequences of the predicted PAO proteins of the three Nicotiana species were aligned and their phylogenetic relation was determined using the Neighbour-Joining algorithm (Figure 2A). Based on this analysis, and the high similarity (98–100%) of the proteins (Figure 2B), most of the *N. tabacum* PAOs could be classified as of potential *N. sylvestris* (1B, 2A, 4A, 4C, 5A, 5C) or *N. tomentosiformis* (1A, 2B, 4B, 4D, 5B, 5D, 5E) origin. This was verified by analysis of gene sequences, including the PAO-coding gene sequences of *N. otophora*, which is also considered a potential ancestor of *N. tabacum* [45,46,47] (Figure 3). This comparison also highlighted that *PAO5*-like sequences are not only the highest in number but are also the most variable among the species. Interestingly, although *N. tabacum* has 14 *PAO* genes, which is exactly the addition of the *N. sylvestris* and *N. tomentosiformis* chromosomes (7 + 7), tobacco has three *PAO2* gene sequences instead of two, and five *PAO5* sequences instead of six, missing the homolog of *PAO5C* of *N. sylvestris* (Figure 2B and Figure 3). The origin of the “extra” PAO2-coding homeolog *NtPAO2C* is uncertain. However, this gene shows a high percent of identity (92.3%) with an *N. otophora* PAO2-coding nucleotide sequence (Figure 3 and Supplementary Figure S2).

### 3.2. Expression Analysis Indicates the Subfunctionalization of Parental PAO-Coding Genes in the Hybrid Tobacco

The hybridization of the parental species of allopolyploid tobacco was a recent event, dating back to 200,000–800,000 years [29,31]. This is why the conservation of the amino acid sequence of the *N. tabacum*, *N. sylvestris*, and *N. tomentosiformis* PAO proteins is very high (95–100%; Figure 2B). To investigate whether, at this high degree of similarity of coded proteins, the parental genes have similar or different functions in the plant’s developmental and environmental responses, the expression pattern of the 14 *N. tabacum* PAO-coding genes was investigated using qRT-PCR.

### 3.3. Organ-Specific Expression of 14 NtPAOs

**Figure 4:**
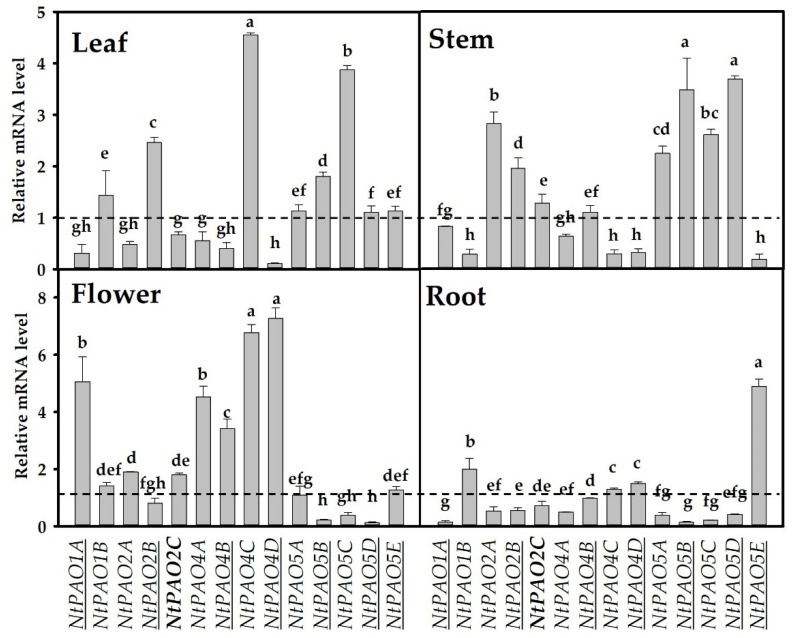
Relative mRNA level of *Nicotiana tabacum PAO* genes in different tobacco organs. Genes of potential *Nicotiana tomentosiformis* origin are underlined, while those of *Nicotiana sylvestris* are not. The *NtPAO2C* gene potentially originating from *Nicotiana otophora* is highlighted in bold. The analysis was performed using quantitative real-time RT-PCR. For gene expression normalization, the mRNA level of NtActin97-like was used. The mRNA level in all four organs was averaged for each gene and served as reference (relative mRNA level = 1 as highlighted by the dashed lines). Data were averaged from three independent biological experiments with three technical replicates each. Standard errors are shown on the columns. The significance of difference between sets of data was determined using one-way analysis of variance (ANOVA) following Duncan’s multiple-range tests; a *p*-value of less than 0.05 was considered significant as indicated by different letters.

Samples were collected from four different tobacco tissues (leaf, stem, flower, and root). The relative mRNA levels of the *N. tabacum PAO* genes in these organs are shown in Figure 4 in reference to their average levels in all organs. It can be stated in general that most of the genes exhibit differentially regulated expression in the various organs and have outstanding relative mRNA levels only in one or two organs. There is no overall correlation between the mRNA levels, or the organ specificity, and the parental origin of the genes. Genes with different parental origins might exhibit similar or contrasting expression in the same organ. While the mRNA levels of *NtPAO1A* are higher in the stem and flowers and lower in the leaves and roots, they show an opposite trend for *NtPAO1B*. The *NtPAO2*A and *NtPAO2B* genes are both strongly expressed in the stem and weakly in the root. In the leaves and flowers, they show somewhat opposite regulation since *NtPAO2*A is more strongly expressed in the flowers than in the leaves, while *NtPAO2B* has a very strong expression in the leaves and only below-average expression in the flowers. *NtPAO2C*, the extra PAO2-coding gene copy of tobacco, has an expression pattern like that of *NtPAO2A*. All *NtPAO4* genes show outstanding expression in the flowers, but *NtPAO4C* also has paramount expression in the leaves as well. Interestingly, in contrast to *NtPAO4C*, *NtPAO4D* is hardly expressed in the leaves, while these two genes have very similar expression levels in the roots, stem, and flowers. Four out of five NtPAO5-coding genes exhibit strong relative expression in the stem and very low in the roots, while the fifth (*NtPAO5E*) has a high expression level in the roots, and low expression in the stem. All PAO5-coding genes are expressed at or below an average level in the flowers, while all of them have at least an average-level expression in the leaf, with *NtPAO5C* exhibiting an outstandingly high mRNA level in this organ. It is also of note that most of the genes have lower-than- or close-to-average expression in the roots, except for *NtPAO5E*, which has the highest expression in this organ.

### 3.4. Expression Changes of Tobacco PAOs in Response to Abiotic Stress Treatments

**Figure 5:**
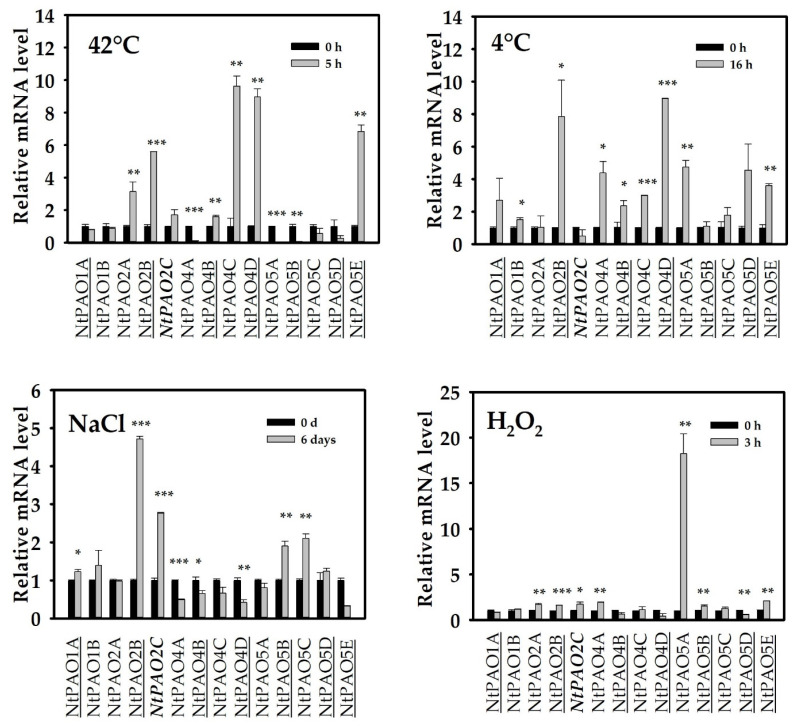
Expression profiles of *NtPAO* genes in tobacco leaves in response to abiotic and stress treatments (heat at 42 °C; cold at 4 °C; 150 mM NaCl, 5 mM H_2_O_2_). Genes of potential *Nicotiana tomentosiformis* origin are underlined, while those of *Nicotiana sylvestris* are not. The *NtPAO2C* gene potentially originating from *Nicotiana otophora* is highlighted in bold. The analysis was performed using quantitative real-time RT-PCR. For gene expression normalization, the mRNA level of the *NtActin97-like* gene was used. The mRNA level of untreated leaves was used as a control (relative mRNA level = 1, shown by black columns). Data were averaged from three independent biological experiments with three technical replicates each. Standard errors are shown on the columns. For significance analysis Student’s *t*-test was used (* *p* ≤ 0.05; ** *p* ≤ 0.01; *** *p* ≤ 0.001).

It has been demonstrated in several studies that the transcript levels of *PAO* genes change upon abiotic stresses, contributing to plant adaptation [8,9,15,21,48,49]. The effect of different abiotic stress treatments (heat, cold, NaCl, H_2_O_2_) on the expression of the 14 tobacco PAO-coding genes was tested to reveal their specific involvement in stress responses. Figure 5 shows that only a handful of *NtPAO* genes responded to the stress treatments but with remarkable specificity. The heat shock of 42 °C induced the *NtPAO2A* and *NtPAO2B* as well as the *NtPAO4C* and *NtPAO4D* pairs of genes with different origins, as well as the *NtPAO5E* gene, which was potentially inherited in the tobacco from *N. tomentosiformis* and has no *N. sylvestris* homeolog in the tobacco genome. Interestingly, heat quickly deregulated the expression of several PAO-coding genes, and the *NtPAO4A* and *NtPAO4B* genes were different in this respect. Neither of the *NtPAO1* genes was responsive to heat. Cold had a more profound effect on *NtPAO* gene expression, augmenting the mRNA level of 8 of the 14 genes, including all 4 NtPAO4-coding ones (Figure 5). One of the *NtPAO2A*-*NtPAO2B*, *NtPAO5A-NtPAO5B*, and *NtPAO5C-NtPAO5D* pairs showed cold induction, while the others did not. This is not in correlation with the genes’ supposed origin. While all NtPAO4-coding genes are activated by cold, all of them are downregulated by salt (Figure 5). The *NtPAO5E* gene shows the same pattern. The *NtPAO2B*, *NtPAO2C*, *NtPAO5B*, and *NtPAO5C* genes exhibit strong induction in response to salt stress, with the other homeologs not showing a similar induction. Several of the *NtPAO* genes strongly responded to H_2_O_2_, among which *NtPAO5A* showed almost a 20-fold induction within three hours (Figure 5). All three *NtPAO2* genes exhibited a positive response, while among the *NtPAO4* ones, only *NtPAO4A* did. The *NtPAO5B* and *NtPAO5E* genes were also induced, while the expression of *NtPAO5B* showed no change, but that of *NtPAO5D* was reduced in response to oxidative stress. It is of note that the NtPAO1-coding genes were hardly responsive to H_2_O_2_, as well as to the other stress treatments.

### 3.5. Expression Responses of Tobacco PAO-Coding Genes in Response to Phytohormones

**Figure 6:**
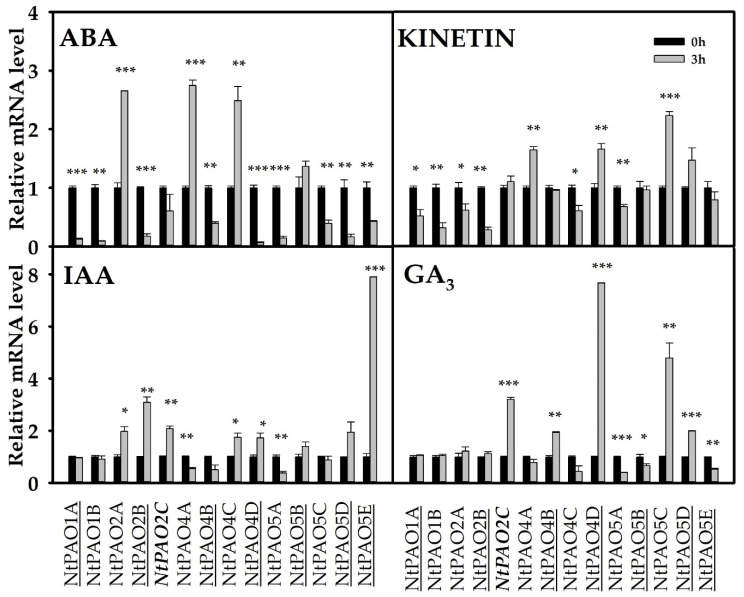
Relative expression of *NtPAO* genes in tobacco leaves in response to various phytohormone (100 μM ABA, 10 μM IAA, 10 μM kinetin, 10 μM GA) treatments. Genes of potential *Nicotiana tomentosiformis* origin are underlined, while those of Nicotiana sylvestris are not. The *NtPAO2C* gene potentially originating from *Nicotiana otophora* is highlighted in bold. For gene expression normalization, the mRNA level of *NtActin97-like* gene was used. The mRNA level of untreated leaves was used as reference (relative mRNA level = 1 shown by the black columns). Data were averaged from three independent biological experiments with three technical replicates each. Standard errors are shown on the columns. For significance analysis, Student’s *t*-test was used (* *p* ≤ 0.05; ** *p* ≤ 0.01; *** *p* ≤ 0.001).

To have an even wider view about the regulation of *NtPAO* genes, different phytohormone treatments were applied to tobacco leaves for a three-hour duration and then changes in the relative transcript levels of *NtPAO*s were examined (Figure 6). The abscisic acid treatment had a profound effect on the *NtPAO* gene expression in the leaves, strongly reducing the expression of 9 out of the 14 genes and elevating approximately three-fold the mRNA level of 3 others. The expression of the NtPAO2- and the NtPAO4-coding genes exhibited contrasting patterns in response to this hormone with induced expression of *NtPAO2A* but reduced expression of *NtPAO2B*, and hardly affected *NtPAO2C*; it elevated expression of *NtPAO4A* and *NtPAO4C* but lowered expression of *NtPAO4B* and *NtPAO4D*. Interestingly, all three ABA-induced genes are of *N. sylvestris* origin. Kinetin influenced *NtPAO* gene expression to a lesser extent. This hormone moderately elevated the mRNA levels of the *NtPAO4A*, *NtPAO4D*, and *NtPAO5C* genes and lowered those of the NtPAO1- and NtPAO2-coding ones (except *NtPAO2C*, which was not affected). The auxin hormone, IAA, strongly induced the expression of *NtPAO5E*, while inhibiting that of *NtPAO5A*. IAA moderately elevated the mRNA levels of the NtPAO2-coding genes, and those of *NtPAO4C* and *NtPAO4D*. GA3 also had a differential effect on the tobacco *PAO* genes. Among the PAO2-coding genes, only *NtPAO2C* responded to the GA3 treatment with an almost four-fold increase. Considering the PAO4-coding genes, the expression of *NtPAO4B* and especially *NtPAO4D* (both of *N. tomentosiformis* origin) was elevated by GA3. The mRNA levels of *NtPAO5A*, *NtPAO5B*, and *NtPAO5E* were reduced while those of *NtPAO5C* and *NtPAO5D* were augmented in response to this hormone.

## 4. Discussion

For many years, tobacco breeders have strived to reduce nicotine and related alkaloid levels, resulting in the development of breeding lines with a low alkaloid content. To reach this goal, scientists, through extensive research based on traditional breeding and molecular biology approaches, have gained insight into the nicotine biosynthetic pathway in tobacco plants and its regulation [50]. It was revealed that tobacco alkaloids are synthesized from amino acid precursors, among others, via polyamine metabolism due to the activity of the enzyme, putrescine n-methyltransferase (PMT) [46,51]. In addition, a crosstalk between polyamine and alkaloid (nicotine) metabolisms has been established: augmented polyamine levels in low-alkaloid tobacco (Burley 21) were shown to impair leaf ripening [52]. An understanding of polyamine metabolism in tobacco might provide clues to achieve a balance between alkaloid and polyamine metabolism in low-nicotine varieties. However, the polyploid nature of tobacco strongly increases the complexity of metabolic regulation, including the metabolism of tri- and tetraamines by the 14 polyamine oxidase (PAO) enzymes.

Here, we provide gene expression data for the members of the *PAO* gene family of tobacco. These data support the diversification of the gene expression patterns of the homeologous genes of tobacco, as well as that of the paralogous genes originating from earlier gene duplication events in ancient angiosperms [26].

### 4.1. The PAO Genes of the Allotetraploid Hybrid N. tabacum Do Not Exactly Match the Parental Sequences

Polyploidy is a major contributor to plant evolution, as verified by recent genomic data revealing that all angiosperms, despite their present genome size and chromosome numbers, have experienced whole-genome duplications multiple times [53]. The Nicotiana genus is a good example, having 76 species, approximately half of which are polyploid [54]. For example, *N. tabacum* is an allotetraploid species (2n = 4x = 48) derived from the cross between the ancestral relatives of *N. sylvestris* (2n = 2x = 24) and *N. tomentosiformis* (2n = 2x = 24), donors of the maternal S-genome and the paternal T-genome, respectively. *N. tabacum* was formed less than a million years ago and thus represents an early stage of diploidization [31]. Despite the changes that have occurred in the repetitive fraction of the *N. tabacum* genome since its formation, most of the genic sequences have remained present in duplicate [54]. In agreement, we identified 14 PAO-coding genes in the tobacco genome (Table 1).

The PAO enzymes are important catalysts of polyamine catabolism, indirectly but significantly influencing plant development and adaptation [20]. Plant PAOs exhibit great diversity in terms of subcellular localization, substrate preference, and reaction products, comprising at least four subfamilies with distinct structural and functional features, one of which predominantly includes the PAOs of monocotyledonous plants [20,26]. The number of *PAO* genes varies among different plant species due to multiple gene duplication and gene loss events that have occurred during evolution [26]. Therefore, the representation of the various PAO subfamilies is often characteristic of a given plant taxon. Arabidopsis has five coding genes for members of the three subfamilies of dicotyledonous plant PAOs, such as the cytoplasmic AtPAO1; the group of peroxisomal AtPAO2, AtPAO3, and AtPAO4 proteins falling into the same subfamily; and the cytoplasmic AtPAO5. The coding genes for AtPAO2- and AtPAO4-like proteins diverged along with the origin of angiosperms [26]. As such, in the scientific literature, angiosperm PAOs are classified into four groups: PAO1 (AtPAO1-like), PAO2 (AtPAO2-like including AtPAO3), PAO4 (AtPAO4-like), and PAO5 (AtPAO5-like).

To define the parental origin of the homeologous tobacco *PAO* genes, the genomic PAO-coding sequences of *N. tabacum*, *N. sylvestris* and *N. tomentosiformis* were identified based on sequence homology with the coded proteins of Arabidopsis PAOs and each other. We identified 14 *N. tabacum,* and 7 *N. sylvestris* and 7 *N. tomentosiformis* PAO-coding gene sequences in agreement with their allopolyploid and diploid nature, respectively. In comparison to Arabidopsis, where the *PAO2* gene was duplicated (designated as *AtPAO2* and *AtPAO3*), in the diploid Nicotiana species, there is one *PAO2* gene, but two *PAO4* and three *PAO5* genes, respectively, indicating different gene duplication or gene loss events taking place after the diversification of the Superrosid and Superasterid clades. The *S. lycopersicum* genome also codes seven different PAO proteins [21] like the diploid *N. sylvestris* and *N. tomentosiformis* species. However, in the Nicotiana species, one of the *PAO5* genes was duplicated, thus having three genes in this group, but it has only one *PAO2* gene, while in *S. lycopersicon,* there are duplicated *PAO2* genes and two *PAO5* genes. This indicates further diversification of the *PAO* gene family during the evolution of Solanaceae. Our study highlights that this diversification continued within the Nicotiana genus. Despite the fact that the parental *N. sylvestris* and *N. tomentosiformis* genomes have 7 PAO genes and their hybrid *N. tabacum* has 14, the tobacco genome was found to code more PAO2-coding and fewer PAO5-coding genes than would be expected from summing up the parental sequences. The sequence similarity indicated the loss of one of the *N. sylvestris* genes for the PAO5-like protein. The origin of the gene coding for the extra PAO2-like protein (NtPAO2C) needs explanation since it has low similarity to the *N. sylvestris* and *N. tomentosiformis PAO2* genes as well. It was found that this gene shows a high percentage of identity (92.3%) with an *N. otophora PAO2*-like nucleotide sequence. Interestingly, the study of another gene family coding for enzymes involved in polyamine metabolism, namely putrescine N-methyltransferases (PMTs), also revealed the presence of *N. otophora* genes in the tobacco genome [46]. Among the five PMT-coding genes, three were found to originate from *N. sylvestris*, one from *N. tomentosiformis*, and one from *N. otophora*. There are indications that *N. othophora* might also have contributed to the *N. tabacum* genome as a third parental species [29,45,46,47]. The evolutionary origin of *N. tabacum* resulting from a cross involving *N. sylvestris* and an introgressed hybrid between *N. tomentosiformis* and *N. otophora* was suggested as a possible scenario. Our finding that the *N. tabacum* genome has an extra *PAO2* gene copy highly similar (above 92% identity) to the *N. othophora* genomic sequences provides further evidence to support the above scenario. It was also suggested that parts of the tobacco genome unmapped by either *N. sylvestris* or *N. tomentosiformis* reads represent introgressions from other Nicotiana species as a consequence of commercial breeding for disease resistance [29].

### 4.2. The Expression Patterns of PAO-Coding Genes in Tobacco Exhibit Diversification in Comparison to Other Dicotyledonous Species

Our gene expression analysis revealed organ specificity as well as diversified hormone and stress responses for the members of the *PAO* gene family in tobacco. This is in agreement with findings in other plant species [14,15,21,48,49,53,54]. However, the expression of gene coding for even the same type of PAO enzymes is highly divergent among species. For example, the PAO1-coding gene of Arabidopsis (*AtPAO1*) has a strong expression peak in flowers [14], unlike the *S. lycopersicon SlPAO1* gene, exhibiting ubiquitous expression at a low level [21]. Considering their expression patterns, *NtPAO1A* resembles *AtPAO1* while *NtPAO1B* resembles *SlPAO1* (Figure 4). The *AtPAO2* and *AtPAO3* genes both have the highest expression in flowers [14]. The tomato PAO2-coding genes *SlPAO2* and *SlPAO3* exhibited high and more or less ubiquitous expression in all organs with peak expression in flowers [21]. However, none of the three PAO2-coding genes of tobacco showed the highest relative expression in flowers (Figure 4). In contrast, all *PAO4* genes have elevated expression in flowers, and low expression in roots (Figure 4), but *AtPAO4* has a strong and rather global expression pattern [14], similar to the *SlPAO4* gene of tomato [21]. While the *PAO5*-type genes in Arabidopsis and tomato exhibit a rather ubiquitous expression in all organs [14,21], the five *PAO5* genes of tobacco have a rather diverse expression pattern (Figure 4).

The involvement of PAO genes in stress responses is well known [8,9,15,20,21,49,54,55,56]. During cold, heat, salt, and H_2_O_2_ stresses, a specific expression pattern of *NtPAO* genes could be observed in the treated leaves (Figure 5). The *NtPAO1A* and *NtPAO1B* genes proved to be the least responsive to the applied stress treatments. In tomato, however, the *SlPAO1* gene exhibited a transient increase in its expression under several stress treatments, including heat, salt and wound [21]. It is of note that in the tomato experiments, samplings were made at earlier time points. If we consider this, only the heat response shows contradictory results; however, the heat shock was applied differently (leaves treated in a water bath versus seedlings treated in a growth chamber) in the two studies.

Involvement of the clade IV genes of Arabidopsis (*AtPAO3*) and tomato (*SlPAO3-5*) was reported in the cold and heat responses [21,23]. In agreement, tobacco *NtPAO2A*, *NtPAO2C*, *NtPAO4C*, and *NtPAO4D* were responsive to heat, while *NtPAO2C* and all *NtPAO4A-D* genes were responsive to cold (Figure 5), suggesting the involvement of these tobacco genes for peroxisomal PAOs in thermoregulation. The regulatory role of PAOs during salt stress has been reported in several studies [8,21,55,56,57]. The Arabidopsis double mutant *atpao1 atpao5*, which is not able to produce none of the cytoplasmic PAO proteins, was found to be tolerant to salinity in contrast to the *atpao2 atpao4* double mutant, which is compromised in the synthesis of peroxisomal isoforms [57]. The Arabidopsis *AtPAO5* gene was found to be strongly induced by salt stress and the loss of its function increased salt stress tolerance according to another study as well [56]. The gene expression of the *NtPAO2B* and *NtPAO2C* genes as well as *NtPAO5B* and *NtPAO5C* were upregulated by salt stress in tobacco (Figure 5), indicating that peroxisomal and cytoplasmic PAOs might also be involved in the salt response, albeit with unknown roles. In tomato, the *SlPAO3* and *SlPAO5* genes also both showed significantly elevated expression in response to salt [21]. Our gene expression data support the view that the various tobacco PAO enzymes have rather diverse roles in stress responses. PAOs have an intricate relationship with H_2_O_2_ as they themselves contribute to H_2_O_2_ production and their gene expression might be either negatively or positively regulated by oxidative stress [20]. The *NtPAO5A* gene uniquely exhibited a very strong and rapid induction in response to H_2_O_2_ stress, which also induced a more moderate but significant increase in the expression of several other tobacco *PAO* genes. (Figure 5). The stress-related downregulation of several *NtPAO* genes (such as *NtPAO4A*, *NtPAO5A*, and *NtPAO5B* using heat or *NtPAO4A*, *NtPAO4B*, and *NtPAO4D* using salt; Figure 5) can help the cells to avoid additional PAO-dependent H_2_O_2_ generation under stress conditions, which themselves cause H_2_O_2_ accumulation [20]. The uncontrolled feedback regulation between the H_2_O_2_-generating PAO and NADPH-oxidase enzymes leads to cell death [8].

One reason for the species-specific gene expression patterns can be that, within the same group, the PAO enzymes have similar biochemical activity, and following gene duplication, the paralogs independently evolved in different species, gaining distinct expression patterns. It also must be considered, however, that the gene expression experiments with the various species were not carried out under the same conditions and therefore might also reflect the sensitivity of the genes to the growth conditions.

### 4.3. The Expression Pattern of Homeologous PAO Genes Exhibit Signs of Subfunctionalization

Analysis of the tobacco leaf transcriptome indicated that a high percentage of the parental genes maintained a conserved expression in tobacco [32]. In approximately 6% of all transcribed genes, the transcripts of only one of the homeologous genes were detected. It remained, however, unknown whether this was the result of gene loss, silencing, or subfunctionalization. Upon expression of both homeologs, only 15% exhibited differential expression, thus providing limited evidence for the subfunctionalization of the parental genes. However, in the absence of a broader gene expression analysis, the significance of subfunctionalization might be underestimated. All 14 *PAO* genes of tobacco were found to be expressed, indicating that their expression is regulated in a way that prevents potential harmful consequences of the increased gene dosage. Considering organ specificity, signs of subfunctionalization could be observed for the homeologous gene pair of *NtPAO1A* and *NtPAO1B*, exhibiting opposite relative gene expression in all organs (Figure 4). In other cases, the preferential or minimal expression of one of the homeologous genes in one specific organ in contrast to its gene pair could be observed, such as the high relative expression of *NtPAO2B* in the leaves in comparison to *NtPAO2A*, the minimal expression of *NtPAO4D* in the same organ in comparison to that of *NtPAO4C*, the strongly root-specific expression of *NtPAO5E* in comparison to all the other four *NtPAO5* genes, and so on (Figure 4). Preferential organ-specific expression of genes dependent on their parental origin could not be observed.

Under stress conditions, some of the homeologous gene pairs exhibited similar regulation, such as *NtPAO1A/NtPAO1B* and *NtPAO4C/NtPAO4D*, while in other cases only one of the homeologous genes showed responsiveness (Figure 5). For example, *NtPAO2B* but not *NtPAO2A* responded to salt and cold, although both reacted similarly to heat and H_2_O_2_. Similarly, *NtPAO4A* and *NtPAO4B* responded in opposite ways to heat, cold, and oxidative stress, but similarly to salt stress. Interestingly, the *NtPAO5A* gene exhibited strong induction in response to H_2_O_2_ and cold stress, but not to salt, and its expression was hardly detectable in the heat-treated leaves. In contrast, the homeologous *NtPAO5B* gene exhibited moderate salt and H_2_O_2_ responsiveness but not cold responsiveness, and was downregulated by heat similarly to *NtPAO5A*. Altogether, the data indicate the diversification of the gene expression of the homeologous genes, which might also be a sign that their subfunctionalization has begun.

This is also supported by their gene expression responses in hormone-treated leaves (Figure 6). The *NtPAO1A* and *NtPAO1B* genes responded the same way to each of the four investigated hormones. ABA upregulated *NtPAO2A*, *NtPAO4A*, and *NtPAO4C* and downregulated *NtPAO2B*, *NtPAO4B*, and *NtPAO4D*. Interestingly, all the upregulated genes are of *N. sylvestris* origin and the downregulated ones are those derived from *N. tomentosiformis* (Figure 6). The *NtPAO5* genes showed a high variability in their hormonal responses without correlation to their origin. The strong auxin-induced expression of the root-expressed *NtPAO5E* gene, with no homeologous counterpart, is of note.

## 5. Conclusions and Outlook

Although the allotetraploid *N. tabacum* is a result of a recent hybridization event at an evolutionary scale, its gene family coding for polyamine oxidases (PAOs) has gone through some rearrangements. One parental *PAO5* gene copy has been lost from its genome while, curiously, it has an extra copy of the *PAO2* gene sequence (*NtPAO2C*). The sequence homology of this latter is a further piece of evidence that *N. otophora* somehow contributed to the formation of *N. tabacum* in addition to the maternal *N. sylvestris* and paternal *N. tomentosiformis* species. Interestingly, *N. otophora* was found to contribute to the polyamine metabolism of tobacco with another gene coding for putrescine n-methyltransferase. Whether it is only a coincidence or the *N. otophora* genes implicated in polyamine metabolism were retained in the tobacco genome due to evolutionary reasons is of future research interest.

The presented gene expression analysis indicates that the gene paralogs formed by gene duplication events during the evolutionary history of angiosperms and the Solanaceae family, as well as the homeologous gene pairs resulting from the hybridization event, often exhibit diverse gene expression patterns, highlighting earlier, as well as ongoing, subfunctionalization of the related genes. The *N. tabacum* genome expresses all 14 PAO-coding genes, and parental-specific gene expression patterns could only be observed in the case of the abscisic-acid-controlled expression of genes controlling peroxisomal PAO isoforms (PAO2 and PAO4 enzymes). Since the subfunctionalization of *N. tabacum PAO* genes is still at an early stage, one can suppose that the biochemical and functional characteristics of the proteins with 98–100% amino acid similarities have not changed yet. However, the knowledge gained about the organ specificity and the stress/hormone responsiveness of *PAO* gene homeologs can help to elaborate strategies to fine-tune polyamine metabolism to improve alkaloid production, development, or stress resilience in tobacco.

## Figures and Tables

**Table 1:** Tobacco polyamine oxidase gene sequences were identified in the genome of the *Nicotiana tabacum* cultivar TN90 [37] ^a^ and the K326 cultivar [29] ^b^, respectively. *Arabidopsis thaliana* PAO sequences were used to query the sequence data derived from the genome sequencing and assembly project PRJNA376174 (*N. tabacum* TN90) and the genome shotgun sequencing project PRJNA319578 (*N. tabacum* K326) using the tblastn algorithm at https://www.ncbi.nlm.nih.gov/datasets/genome/?taxon=4097 (last accessed on 1 September 2023). bp—base pair; aa—aminoacid.

Gene Accession ^a^	Gene Accession ^b^	Scaffold Accession (Gene Position)	mRNA Accession	mRNA Size (bp)	Protein Accession	Protein Size (aa)	Gene Annotation	Gene Name
LOC107832568	Nitab4.5_0000707g0200.1	NW_015864215.1 (20974..27671)	NM_001326282.2	1888	NP_001313211.1	495	polyamine oxidase 1	*NtPAO1A*
LOC107788770	Nitab4.5_0008441g0030.1	NW_015916267.1 (46836..53239)	XM_016610479.1	1801	XP_016465965.1	447	polyamine oxidase 1-like	*NtPAO1B*
LOC107762338	Nitab4.5_0002015g0070.1	NW_015876609.1 (111786..118428,	XM_016580681.1	2235	XP_016436167.1	488	probable polyamine oxidase 2	*NtPAO2A*
LOC107775961	Nitab4.5_0004862g0070.1	NW_015901231.1 (12262..17390)	XM_016595768.1	2295	XP_016451254.1	488	probable polyamine oxidase 2	*NtPAO2B*
LOC107799822	Nitab4.5_0007665g0030.1	NW_015931733.1 (28305..34373,	XM_016622969.1	2300	XP_016478455.1	490	probable polyamine oxidase 2	*NtPAO2C*
LOC107800087	Nitab4.5_0001374g0220.1	NW_015932137.1 (603..6066,	XM_016623232.1	2199	XP_016478718.1	495	probable polyamine oxidase 4	*NtPAO4A*
LOC107775720	Nitab4.5_0000091g0280.1	NW_015900952.1 (12745..17683	XM_016595473.1	1492	XP_016450959.1	416	probable polyamine oxidase 4	*NtPAO4B*
LOC107761719	Nitab4.5_0003412g0030.1	NW_015874368.1 (104479..111727	XM_016579980.1	2802	XP_016435466.1	495	probable polyamine oxidase 4	*NtPAO4_C*
LOC107812697	Nitab4.5_0000483g0140.1	NW_015954488.1 (6193..10774)	XM_016637848.1	2094	XP_016493334.1	399	probable polyamine oxidase 4	*NtPAO4D*
LOC107813795	Nitab4.5_0004978g0010.1	NW_015789106.1 (85803..87734)	XM_016639103.1	1932	XP_016494589.1	536	probable polyamine oxidase 5	*NtPAO5A*
LOC107765565	Nitab4.5_0016456g0010.1	NW_015794575.1 (4084..5978,	XM_016584233.1	1895	XP_016439719.1	531	probable polyamine oxidase 5	*NtPAO5B*
LOC107791914	Nitab4.5_0003310g0050.1	NW_015920426.1 (26034..28297,	XM_016614062.1	2264	XP_016469548.1	533	probable polyamine oxidase 5	*NtPAO5C*
LOC107767845	Nitab4.5_0000095g0080.1	NW_015887926.1 (167021..169486	XM_016586944.1	2466	XP_016442430.1	539	probable polyamine oxidase 5	*NtPAO5D*
LOC107778196	Nitab4.5_0008523g0010.1	NW_015903755.1 (63036..64820)	XM_016598410.1	1785	XP_016453896.1	543	probable polyamine oxidase 5	*NtPAO5E*

## Data Availability

No new data were created.

## Supplementary Materials

The following supporting information can be downloaded at https://www.mdpi.com/article/10.3390/genes14112025/s1, Figure S1: Amino acid sequence identity of five Arabidopsis thaliana and fourteen Nicotiana tabacum POLYAMINE OXIDASE (PAO) proteins; Figure S2: Phylogenetic comparison of Nicotiana PAO2-coding gene sequences; Table S1: Primer sequences used to amplify the transcripts of the PAO-coding and the reference genes; Table S2: The five Arabidopsis PAO genes with their gene and protein identifiers; Table S3: Accessions of the carrying genomic scaffolds, PAO-coding genes, and predicted PAO proteins of *N. sylvestris* TW136 (NSYLV), *N. tomentosiformis* (NTOM), and *N. otophora* (NOTO).

## References

[1] Tiburcio A.F., Altabella T., Bitrián M., Alcázar R. (2014). The Roles of Polyamines during the Lifespan of Plants: From Development to Stress. Planta.

[2] Benkő P., Jee S., Kaszler N., Fehér A., Gémes K. (2020). Polyamines Treatment during Pollen Germination and Pollen Tube Elongation in Tobacco Modulate Reactive Oxygen Species and Nitric Oxide Homeostasis. J. Plant Physiol..

[3] Kaszler N., Benkő P., Bernula D., Szepesi Á., Fehér A., Gémes K., Benk P., Bernula D., Szepesi Á., Fehér A. (2021). Polyamine Metabolism Is Involved in the Direct Regeneration of Shoots from Arabidopsis Lateral Root Primordia. Plants.

[4] Fortes A.M., Agudelo-Romero P. (2018). Polyamine Metabolism in Climacteric and Non-Climacteric Fruit Ripening.

[5] Cai G., Sobieszczuk-Nowicka E., Aloisi I., Fattorini L., Serafini-Fracassini D., Del Duca S. (2015). Polyamines Are Common Players in Different Facets of Plant Programmed Cell Death. Amino Acids.

[6] Groppa M.D., Benavides M.P. (2008). Polyamines and Abiotic Stress: Recent Advances. Amino Acids.

[7] Moschou P.N., Paschalidis K.A., Roubelakis-Angelakis K.A. (2008). Plant Polyamine Catabolism. Plant Signal. Behav..

[8] Gémes K., Kim Y.J., Park K.Y., Moschou P.N., Andronis E., Valassaki C., Roussis A., Roubelakis-Angelakis K.A. (2016). An NADPH-Oxidase/Polyamine Oxidase Feedback Loop Controls Oxidative Burst under Salinity. Plant Physiol..

[9] Gémes K., Mellidou Ι., Karamanoli K., Beris D., Park K.Y., Matsi T., Haralampidis K., Constantinidou H.I., Roubelakis-Angelakis K.A. (2017). Deregulation of Apoplastic Polyamine Oxidase Affects Development and Salt Response of Tobacco Plants. J. Plant Physiol..

[10] Ebeed H.T., Hassan N.M., Aljarani A.M. (2017). Exogenous Applications of Polyamines Modulate Drought Responses in Wheat through Osmolytes Accumulation, Increasing Free Polyamine Levels and Regulation of Polyamine Biosynthetic Genes. Plant Physiol. Biochem..

[11] Takahashi T. (2020). Plant Polyamines. Plants.

[12] Smith T.A. (1985). Polyamines. Annu. Rev. Plant Physiol..

[13] Kusano T., Berberich T., Tateda C., Takahashi Y. (2008). Polyamines: Essential Factors for Growth and Survival. Planta.

[14] Takahashi Y., Cong R., Sagor G.H.M.M.H.M.M., Niitsu M., Berberich T., Kusano T. (2010). Characterization of Five Polyamine Oxidase Isoforms in Arabidopsis Thaliana. Plant Cell Rep..

[15] Sagor G.H.M., Inoue M., Kusano T., Berberich T. (2021). Expression Profile of Seven Polyamine Oxidase Genes in Rice (*Oryza sativa*) in Response to Abiotic Stresses, Phytohormones and Polyamines. Physiol. Mol. Biol. Plants.

[16] Fujita M., Shinozaki K. (2015). Polyamine Transport Systems in Plants. Polyamines: A Universal Molecular Nexus for Growth, Survival, and Specialized Metabolism.

[17] Liu T., Kim D.W., Niitsu M., Berberich T., Kusano T. (2014). Oryza Sativa Polyamine Oxidase 1 Back-Converts Tetraamines, Spermine and Thermospermine, to Spermidine. Plant Cell Rep..

[18] Bagni N., Tassoni A. (2001). Biosynthesis, Oxidation and Conjugation of Aliphatic Polyamines in Higher Plants. Amino Acids.

[19] Cona A., Rea G., Angelini R., Federico R., Tavladoraki P. (2006). Functions of Amine Oxidases in Plant Development and Defence. Trends Plant Sci..

[20] Benkő P., Gémes K., Fehér A. (2022). Polyamine Oxidase-Generated Reactive Oxygen Species in Plant Development and Adaptation: The Polyamine Oxidase—NADPH Oxidase Nexus. Antioxidants.

[21] Hao Y., Huang B., Jia D., Mann T., Jiang X., Qiu Y., Niitsu M., Berberich T., Kusano T., Liu T. (2018). Identification of Seven Polyamine Oxidase Genes in Tomato (*Solanum lycopersicum* L.) and Their Expression Profiles under Physiological and Various Stress Conditions. J. Plant Physiol..

[22] Wang W., Paschalidis K., Feng J.-C.C., Song J., Liu J.-H.H. (2019). Polyamine Catabolism in Plants: A Universal Process with Diverse Functions. Front. Plant Sci..

[23] Toumi I., Pagoulatou M.G., Margaritopoulou T., Milioni D., Roubelakis-Angelakis K.A. (2019). Genetically Modified Heat Shock Protein90s and Polyamine Oxidases in Arabidopsis Reveal Their Interaction under Heat Stress Affecting Polyamine Acetylation, Oxidation and Homeostasis of Reactive Oxygen Species. Plants.

[24] Mellidou I., Karamanoli K., Beris D., Haralampidis K., Constantinidou H.-I.A., Roubelakis-Angelakis K.A. (2017). Underexpression of Apoplastic Polyamine Oxidase Improves Thermotolerance in Nicotiana Tabacum. J. Plant Physiol..

[25] Konstantinos P.A., Imene T., Panagiotis M.N., Roubelakis-Angelakis K.A. (2010). ABA-Dependent Amine Oxidases-Derived H2O2 Affects Stomata Conductance. Plant Signal. Behav..

[26] Bordenave C.D., Granados Mendoza C., Jiménez Bremont J.F., Gárriz A., Rodríguez A.A. (2019). Defining Novel Plant Polyamine Oxidase Subfamilies through Molecular Modeling and Sequence Analysis. BMC Evol. Biol..

[27] Kim D.W., Watanabe K., Murayama C., Izawa S., Niitsu M., Michael A.J., Berberich T., Kusano T. (2014). Polyamine Oxidase5 Regulates Arabidopsis Growth through Thermospermine Oxidase Activity. Plant Physiol..

[28] Ono Y., Kim D.W., Watanabe K., Sasaki A., Niitsu M., Berberich T., Kusano T., Takahashi Y. (2012). Constitutively and Highly Expressed Oryza Sativa Polyamine Oxidases Localize in Peroxisomes and Catalyze Polyamine Back Conversion. Amino Acids.

[29] Edwards K.D., Fernandez-Pozo N., Drake-Stowe K., Humphry M., Evans A.D., Bombarely A., Allen F., Hurst R., White B., Kernodle S.P. (2017). A Reference Genome for Nicotiana Tabacum Enables Map-Based Cloning of Homeologous Loci Implicated in Nitrogen Utilization Efficiency. BMC Genom..

[30] Magadum S., Banerjee U., Murugan P., Gangapur D., Ravikesavan R. (2013). Gene Duplication as a Major Force in Evolution. J. Genet..

[31] Clarkson J.J., Dodsworth S., Chase M.W. (2017). Time-Calibrated Phylogenetic Trees Establish a Lag between Polyploidisation and Diversification in Nicotiana (Solanaceae). Plant. Syst. Evol..

[32] Bombarely A., Edwards K.D., Sanchez-Tamburrino J., Mueller L.A. (2012). Deciphering the Complex Leaf Transcriptome of the Allotetraploid Species Nicotiana Tabacum: A Phylogenomic Perspective. BMC Genom..

[33] Murashige T., Skoog F. (1962). A Revised Medium for Rapid Growth and Bio Assays with Tobacco Tissue Cultures. Physiol. Plant..

[34] Ye J., Coulouris G., Zaretskaya I., Cutcutache I., Rozen S., Madden T.L. (2012). Primer-BLAST: A Tool to Design Target-Specific Primers for Polymerase Chain Reaction. BMC Bioinform..

[35] Sierro N., Battey J.N.D., Ouadi S., Bakaher N., Bovet L., Willig A., Goepfert S., Peitsch M.C., Ivanov N.V. (2014). The Tobacco Genome Sequence and Its Comparison with Those of Tomato and Potato. Nat. Commun..

[36] Johnson M., Zaretskaya I., Raytselis Y., Merezhuk Y., McGinnis S., Madden T.L. (2008). NCBI BLAST: A Better Web Interface. Nucleic Acids Res..

[37] Bailey T.L., Johnson J., Grant C.E., Noble W.S. (2015). The MEME Suite. Nucleic Acids Res..

[38] Darzentas N. (2010). Circoletto: Visualizing Sequence Similarity with Circos. Bioinformatics.

[39] Tamura K., Stecher G., Kumar S. (2021). MEGA11: Molecular Evolutionary Genetics Analysis Version 11. Mol. Biol. Evol..

[40] Larkin M.A., Blackshields G., Brown N.P., Chenna R., McGettigan P.A., McWilliam H., Valentin F., Wallace I.M., Wilm A., Lopez R. (2007). Clustal W and Clustal X Version 2.0. Bioinformatics.

[41] Saitou N., Nei M. (1987). The Neighbor-Joining Method: A New Method for Reconstructing Phylogenetic Trees. Mol. Biol. Evol..

[42] Zuckerland E., Pauling L., Bryson V., Vogel H.J. (1965). Evolutionary Divergence and Convergence in Proteins. Evolving Genes and Proteins.

[43] Horton P., Park K.-J., Obayashi T., Fujita N., Harada H., Adams-Collier C.J., Nakai K. (2007). WoLF PSORT: Protein Localization Predictor. Nucleic Acids Res..

[44] Sierro N., Battey J.N., Ouadi S., Bovet L., Goepfert S., Bakaher N., Peitsch M.C., Ivanov N.V. (2013). Reference Genomes and Transcriptomes of Nicotiana Sylvestris and Nicotiana Tomentosiformis. Genome Biol..

[45] Kenton A., Parokonny A.S., Gleba Y.Y., Bennett M.D. (1993). Characterization of the *Nicotiana tabacum* L. Genome by Molecular Cytogenetics. Mol. Gen. Genet..

[46] Riechers D.E., Timko M.P. (1999). Structure and Expression of the Gene Family Encoding Putrescine N-Methyltransferase in Nicotiana Tabacum: New Clues to the Evolutionary Origin of Cultivated Tobacco. Plant. Mol. Biol..

[47] Ren N., Timko M.P. (2001). AFLP Analysis of Genetic Polymorphism and Evolutionary Relationships among Cultivated and Wild *Nicotiana* Species. Genome.

[48] Li M., Lu J., Tao M., Li M., Yang H., Xia E., Chen Q., Wan X. (2020). Genome-Wide Identification of Seven Polyamine Oxidase Genes in *Camellia sinensis* (L.) and Their Expression Patterns Under Various Abiotic Stresses. Front. Plant Sci..

[49] Zhang J., Liang L., Xiao J., Xie Y., Zhu L., Xue X., Xu L., Zhou P., Ran J., Huang Z. (2022). Genome-Wide Identification of Polyamine Oxidase (PAO) Family Genes: Roles of CaPAO2 and CaPAO4 in the Cold Tolerance of Pepper (*Capsicum annuum* L.). Int. J. Mol. Sci..

[50] Shoji T., Moriyama K., Sierro N., Ouadi S., Ivanov N.V., Hashimoto T., Saito K. (2022). Natural and Induced Variations in Transcriptional Regulator Genes Result in Low-Nicotine Phenotypes in Tobacco. Plant J..

[51] Dewey R.E., Xie J. (2013). Molecular Genetics of Alkaloid Biosynthesis in Nicotiana Tabacum. Phytochemistry.

[52] Nölke G., Chudobova I., Houdelet M., Volke D., Lusso M., Frederick J., Kudithipudi C., Shen Y., Warek U., Strickland J.A. (2021). Impact of Nicotine Pathway Downregulation on Polyamine Biosynthesis and Leaf Ripening in Tobacco. Plant Direct.

[53] Fincato P., Moschou P.N., Ahou A., Angelini R., Roubelakis-Angelakis K.A., Federico R., Tavladoraki P. (2012). The Members of Arabidopsis Thaliana PAO Gene Family Exhibit Distinct Tissue- and Organ-Specific Expression Pattern during Seedling Growth and Flower Development. Amino Acids.

[54] Samanta I., Roy P.C., Das E., Mishra S., Chowdhary G. (2023). Plant Peroxisomal Polyamine Oxidase: A Ubiquitous Enzyme Involved in Abiotic Stress Tolerance. Plants.

[55] Wang W., Liu J.-H. (2016). CsPAO4 of Citrus Sinensis Functions in Polyamine Terminal Catabolism and Inhibits Plant Growth under Salt Stress. Sci. Rep..

[56] Zarza X., Atanasov K.E., Marco F., Arbona V., Carrasco P., Kopka J., Fotopoulos V., Munnik T., Gómez-Cadenas A., Tiburcio A.F. (2017). Polyamine Oxidase 5 Loss-of-Function Mutations in Arabidopsis Thaliana Trigger Metabolic and Transcriptional Reprogramming and Promote Salt Stress Tolerance. Plant Cell Environ..

[57] Sagor G.H.M., Zhang S., Kojima S., Simm S., Berberich T., Kusano T. (2016). Reducing Cytoplasmic Polyamine Oxidase Activity in Arabidopsis Increases Salt and Drought Tolerance by Reducing Reactive Oxygen Species Production and Increasing Defense Gene Expression. Front. Plant Sci..

