# Supplementary material for: Subfunctionalization of Parental Polyamine Oxidase (PAO) Genes in the Allopolyploid Tobacco Nicotiana tabacum (L.)
Source: Genes (Basel). 2023 Oct 30;14(11):2025. doi: 10.3390/genes14112025 (PMC10671180; doi:10.3390/genes14112025)
Supplement: Supplementary file 1 [file genes-14-02025-s001.zip › Suppl fig 1.pptx]

## Slide 1
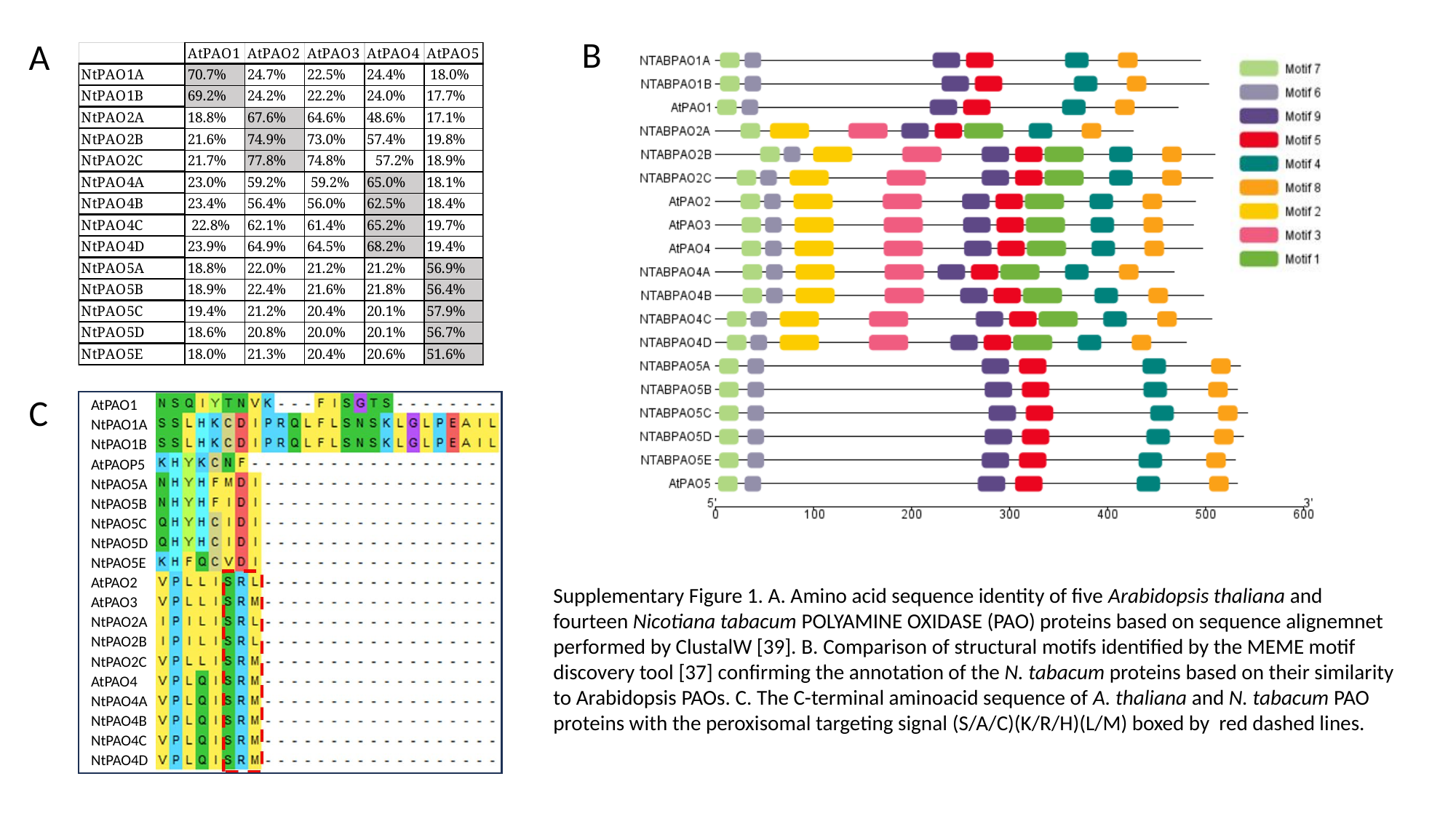

B
A
C
AtPAO1
NtPAO1A
NtPAO1B
AtPAOP5
NtPAO5A
NtPAO5B
NtPAO5C
NtPAO5D
NtPAO5E
AtPAO2
AtPAO3
NtPAO2A
NtPAO2B
NtPAO2C
AtPAO4
NtPAO4A
NtPAO4B
NtPAO4C
NtPAO4D
Supplementary Figure 1. A. Amino acid sequence identity of five Arabidopsis thaliana and fourteen Nicotiana tabacum POLYAMINE OXIDASE (PAO) proteins based on sequence alignemnet performed by ClustalW [39]. B. Comparison of structural motifs identified by the MEME motif discovery tool [37] confirming the annotation of the N. tabacum proteins based on their similarity to Arabidopsis PAOs. C. The C-terminal aminoacid sequence of A. thaliana and N. tabacum PAO proteins with the peroxisomal targeting signal (S/A/C)(K/R/H)(L/M) boxed by red dashed lines.
