# Supplementary material for: Subfunctionalization of Parental Polyamine Oxidase (PAO) Genes in the Allopolyploid Tobacco Nicotiana tabacum (L.)
Source: Genes (Basel). 2023 Oct 30;14(11):2025. doi: 10.3390/genes14112025 (PMC10671180; doi:10.3390/genes14112025)
Supplement: Supplementary file 1 [file genes-14-02025-s001.zip › Suppl fig 2.pptx]

## Slide 1
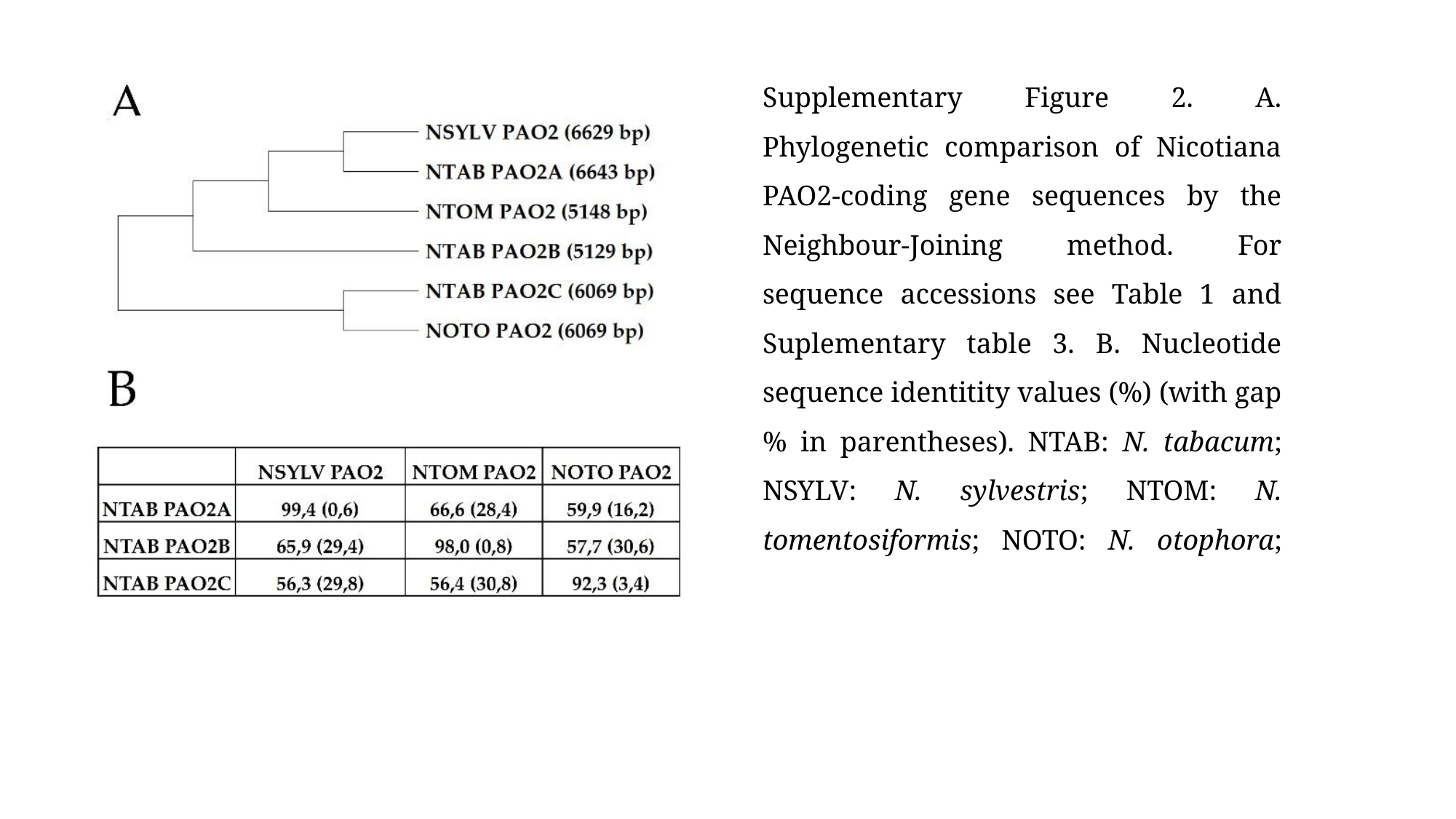

Supplementary Figure 2. A. Phylogenetic comparison of Nicotiana PAO2-coding gene sequences by the Neighbour-Joining method. For sequence accessions see Table 1 and Suplementary table 3. B. Nucleotide sequence identitity values (%) (with gap% in parentheses). NTAB: N. tabacum; NSYLV: N. sylvestris; NTOM: N. tomentosiformis; NOTO: N. otophora;
