# Supplementary material for: Subfunctionalization of Parental Polyamine Oxidase (PAO) Genes in the Allopolyploid Tobacco Nicotiana tabacum (L.)
Source: Genes (Basel). 2023 Oct 30;14(11):2025. doi: 10.3390/genes14112025 (PMC10671180; doi:10.3390/genes14112025)
Supplement: Supplementary file 1 [file genes-14-02025-s001.zip › supplementary Table S1.pdf]

Short name	Gene Accession	mRNA accesion	orientation	Primer sequence 5'->3'
**NtPAO1_A**	LOC107832568	XM_016610479.1	forward	CGGCGTATTGGTGTCAACAG
			reverse	TGTGAAGGAAATGAGGTCGCT
**NtPAO1_B**	LOC107788770	XM_016610479.1	forward	TCCCCTCGGCCTCTTTTATG
			reverse	TATGACGACAGAGCAGCGAC
**NtPAO2_A**	LOC107762338	XM_016580681.1	forward	CTTCCTAATGCGACTGCTCCA
			reverse	CATGGGGCTTCCCAACTGTA
**NtPAO2_B**	LOC107775961	XM_016595768.1	forward	CACGTCTACGTAGGAAACACCA
			reverse	TTGGGAAGACGAAGGGGAGA
**NtPAO2_C**	LOC107799822	XM_016622969.1	forward	CCCAGAGTTCTCTTAATTCCCCT
			reverse	CTCTCTCCGGATCAAACAATGGA
**NtPAO4_A**	LOC107800087	XM_016623232.1	forward	TCCCCTCCAGATTTCCAGGAT
			reverse	GCACGAGTGCTACAACTTCATT
**NtPAO4_B**	LOC107775720	XM_016595473.1	forward	GTTTCCTAATGCACCTGCCC
			reverse	TCTGTTCCCCAGCGTGATAC
**NtPAO4_C**	LOC107761719	XM_016579980.1	forward	CCCCGATGCTACTAAGCCTG
			reverse	CCAATGGTGCTCGAAGCCTA
**NtPAO4_D**	LOC107812697	XM_016637848.1	forward	TCCACAAGGCAACAGGACAT
			reverse	AAATTAGCATGGGTGCAACACT
**NtPAO5_A**	LOC107813795	XM_016639103.1	forward	CAATCTCAAGGCTTGGTTTTGGT
			reverse	TGTTCTCCTCATCCACCAAGGA
**NtPAO5_B**	LOC107765565	XM_016584233.1	forward	TCTGCCACCTGGTTTGATCC
			reverse	AATGAACCATCAGCCTGCCA
**NtPAO5_C**	LOC107791914	XM_016614062.1	forward	TCTGATTGTTGAGGGGGTTGT
			reverse	CACCACTGTTACATACACACACATA
**NtPAO5_D**	LOC107767845	XM_016586944.1	forward	GTCTGTGATTGTTGAGGGGGT
			reverse	CACACACACATAGCTGTCCCA
**NtPAO5_E**	LOC107778196	XM_016598410.1	forward	GGAATTCGCGACGACTTTGC
			reverse	TCATCCCCTCATAGCCACATTC
**Actin97-like**	LOC107804820	XM_016628756.1	forward	CAGTGTCTGGATTGGAGGTTC
			reverse	ATCTGCTGGAAGGTGCTGAG
**L25**	L18908	XM_016628756.1	forward	CACCCTTGTTTTCATTGTGG
			reverse	ATGCTTTCTTCGTCCCATCA

Supplementary Table S1. Primer sequences used to amplify the transcripts of the PAO-coding and the reference genes (*Actin97-like* and *L25*) of tobacco.
