# Supplementary material for: Subfunctionalization of Parental Polyamine Oxidase (PAO) Genes in the Allopolyploid Tobacco Nicotiana tabacum (L.)
Source: Genes (Basel). 2023 Oct 30;14(11):2025. doi: 10.3390/genes14112025 (PMC10671180; doi:10.3390/genes14112025)
Supplement: Supplementary file 1 [file genes-14-02025-s001.zip › supplementary Table S2.pdf]

**gene name**	**gene id**	**protein id**
AtPAO1	AT5G13700	NP_196874.1
AtPAO2	AT2G43020	NP_181830.1
AtPAO3	AT3G59050	NP_191464.1
AtPAO4	AT1G65840	NP_176759.1
AtPAO5	AT4G29720	NP_194701.1

Supplementary Table S2. The five Arabidopsis PAO genes with their gene and protein identifiers (https://www.arabidopsis.org/; last accessed 01.09.2023).
