# Supplementary material for: Subfunctionalization of Parental Polyamine Oxidase (PAO) Genes in the Allopolyploid Tobacco Nicotiana tabacum (L.)
Source: Genes (Basel). 2023 Oct 30;14(11):2025. doi: 10.3390/genes14112025 (PMC10671180; doi:10.3390/genes14112025)
Supplement: Supplementary file 1 [file genes-14-02025-s001.zip › supplementary Table S3.pdf]

Name/Accession	Scaffold	Gene	Protein
NSYLV_PAO1	NW_009358137.1	LOC104211234	XP_009758563
NSYLV_PAO2	NW_009580520.1	LOC104226820	XP_009777198
NSYLV_PAO4A	NW_009360037.1	LOC104213745	XP_009761595
NSYLV_PAO4B	NW_009385493.1	LOC104230337	XP_009781420
NSYLV_PAO5A	NW_009510987.1	LOC104242553	XP_009795930
NSYLV_PAO5B	NW_009429758.1	LOC104235015	XP_009786985
NSYLV_PAO5C	NW_009533522.1	LOC104214356	XP_009762313
NTOM_PAO1	NW_008895960.1	LOC104104873	XP_009611355
NTOM_PAO2	NW_008924628.1	LOC104086117	XP_009588592
NTOM_PAO4A	NW_008837126.1	LOC104107681	XP_033514967
NTOM_PAO4B	NW_008948018.1	LOC104093043	XP_009597054
NTOM_PAO5A	NW_008839590.1	LOC104111189	XP_009619131
NTOM_PAO5B	NW_008977363.1	LOC104098345	XP_009603352
NTOM_PAO5C	NW_008947713.1	LOC104092977	XP_009596969

Supplementary Table S3. Accessions of the carrying genomic scaffolds, PAO-coding genes, and predicted PAO proteins of *N. sylvestris* TW136 (NSYLV), and *N. tomentosiformis* (NTOM) are shown.
